# Clinically informed preoperative risk stratification for MRI-defined non gross-total resection in nonfunctioning pituitary neuroendocrine tumors: a single-center internal validation study

**DOI:** 10.3389/fphys.2026.1948507

**Published:** 2026-09-07

**Authors:** Shuting Yang, Haosen Jiao, Xuan Zheng, Chengbin Duan, Weiyu Hu, Dimin Zhu, Jinping Chen, Zongming Wang, Wenli Chen

**Affiliations:** Department of Neurosurgery, Sixth Affiliated Hospital of Sun Yat-sen University, Guangzhou, China

**Keywords:** NF-PitNET, non-gross-total resection, pituitary neuroendocrine tumor, pituitary surgery, risk stratification

## Abstract

**Introduction:**

Preoperative estimation of non-gross-total resection (non-GTR), as defined on early postoperative magnetic resonance imaging (MRI), may support patient counseling and postoperative surveillance planning after surgery for nonfunctioning pituitary neuroendocrine tumors (NF-PitNETs). We evaluated whether a clinically informed, interpretable preoperative modeling strategy improved risk estimation compared with a reference strategy.

**Methods:**

We retrospectively analyzed 354 first-surgery patients from a single center with a documented early postoperative MRI-based resection-status endpoint; 57 (16.1%) had non-GTR. A prespecified clinically informed logistic strategy extended a reference model based on routine preoperative clinical and MRI variables by adding transformed terms representing nonlinear tumor burden and invasion severity. Models underwent stratified repeated nested cross-validation with five outer folds repeated 20 times and four-fold inner tuning. Performance was evaluated using patient-level averaged held-out predictions.

**Results:**

Under model-specific tuning strategies, the clinically informed strategy showed little change in discrimination compared with the reference strategy (area under the receiver operating characteristic curve, 0.8054 vs. 0.8022; area under the precision-recall curve, 0.4603 vs. 0.4534), but had a lower Brier score (0.1100 vs. 0.1480) and calibration closer to ideal (intercept, 0.0256 vs. −0.3778; slope, 1.0209 vs. 2.5233). It also showed greater model-based net benefit across an exploratory threshold range of 0.05–0.50 and separated cohort-derived tertiles with observed non-GTR rates of 4.2%, 8.5%, and 35.6%. Postoperative pathological variables provided little incremental value.

**Discussion:**

These findings represent single-center internal validation of complete modeling strategies. Because the strategies differed in both predictor representation and tuning objective, the calibration difference cannot be attributed to the transformed predictors alone. Independent external validation using standardized postoperative MRI, together with uniform-tuning and reduced-model sensitivity analyses, is required before application to counseling or surveillance planning.

## 1 Introduction

Residual disease after surgery for clinically nonfunctioning pituitary tumors remains clinically important because visible postoperative residual tumor carries the highest near-term regrowth risk, whereas patients without residual tumor on early postoperative imaging have a much lower short-term recurrence burden (Maletkovic et al., 2019; Gerges et al., 2021). Contemporary management reviews likewise emphasize that extent of resection shapes postoperative surveillance, adjuvant decision-making, and patient counseling in non-functioning pituitary adenomas (Esposito et al., 2019). That downstream consequence makes preoperative estimation of early residual or non-gross-total-resection risk relevant for counseling, operative expectations, and early follow-up planning, even before any postoperative imaging result is known.

The recent WHO classification uses the term pituitary neuroendocrine tumor (PitNET) for adenohypophyseal tumors while recognizing their historical pituitary adenoma nomenclature (Asa et al., 2022). In parallel, the preoperative pituitary surgery literature has mostly approached the problem as one of structural resectability. Single-center and multicenter surgical series consistently identify tumor size, Knosp grade, and invasion burden as dominant determinants of gross total resection feasibility (Juraschka et al., 2014; Perez-Lopez et al., 2021). That line has also produced compact grading systems such as the Zurich Pituitary Score, HACKD score, and the original-to-modified TRANSSPHER grading line, which are clinically legible but principally target surgical complexity or gross-total-resection likelihood rather than calibrated individual residual-risk estimation (Mooney et al., 2019; Staartjes et al., 2020; Castle-Kirszbaum et al., 2022; Fiore et al., 2025). More recent anatomy-limit and landmark-based models continue that same route with predictive scales and nomograms built from preoperative MRI morphology, including newer NFPA-specific radiological scores that combine Knosp with SIPAP extension patterns, giant nonfunctioning PitNET predictive modeling from structured multi-planar variables, automated grading, and new multivariate morphologic models for giant adenomas; even so, the target usually remains resection feasibility or surgical complexity rather than prespecified residual-risk estimation for clinical use (Martorell-Llobregat et al., 2023; Shen et al., 2024; Da Mutten et al., 2025; Giardini et al., 2025; Patel et al., 2025a; Ozaydin et al., 2026).

Recent prediction studies have explored a different direction. Machine-learning pilots have been used to predict early postoperative outcomes or gross-total-resection classification after pituitary surgery (Hollon et al., 2018; Staartjes et al., 2018), and a later Zurich-Bologna study externally validated preoperative pituitary-surgery prediction models across multiple postoperative endpoints (Zanier et al., 2023). The broader literature now also includes radiomic GTR/consistency modeling and CNN-based extent-of-resection regression from preoperative MRI, while a 2025 systematic review/meta-analysis suggests that remission prediction after pituitary surgery is also entering the ML literature, albeit on endpoint-mixed postoperative cohorts rather than prespecified preoperative residual-risk populations (Cerny et al., 2025; Mohammadzadeh et al., 2025; Patel et al., 2025b). A separate 2026 meta-analysis suggests that ML-based preoperative consistency prediction can look strong in aggregate but still depends on standardized imaging and external validation before translation (Hajikarimloo et al., 2026). At the same time, a pituitary-surgery ML review found variable reporting quality, frequent omission of calibration reporting, and very sparse external validation, while the more recent radiomics review also found marked methodological heterogeneity and limited external validation (Rech et al., 2023; Agosti et al., 2025).

Existing preoperative pituitary prediction studies span a continuum from interpretable structural feasibility grades to higher-capacity imaging and machine-learning models, but most focus on resection-feasibility classification, score automation, or postoperative-context proxies rather than calibrated probabilities of an early postoperative MRI-defined resection endpoint. The present study addresses a narrower clinical question: whether a clinically informed, interpretable preoperative modeling strategy can improve calibration, model-based net benefit, and descriptive risk stratification for MRI-defined non-GTR when evaluated against a preserved reference strategy in the same single-center cohort and outer validation splits. Throughout this report, MRI-defined non-GTR is the preferred primary term and denotes enhancing residual tumor on the institutionally adjudicated early postoperative MRI; it is not a long-term residual-growth or recurrence outcome.

Because the intended future use is preoperative counseling and perioperative planning, discrimination alone is insufficient. Calibration is essential for valid risk communication and decision support (Van Calster et al., 2016, 2019), and decision-curve analysis quantifies model-based net benefit across threshold probabilities rather than only ranking patients (Vickers and Elkin, 2006). Transparent prediction-model reporting and risk-of-bias appraisal also require explicit validation design, intended use, and applicability, particularly when interpretable regression and machine-learning comparators are presented together (Collins et al., 2015, 2024; Moons et al., 2025). We therefore compared a prespecified clinically informed preoperative strategy with the reference preoperative strategy while preserving one frozen cohort and a shared outer internal-validation design.

## 2 Materials and methods

### 2.1 Study design, cohort, and outcome definition

**Figure 1:**
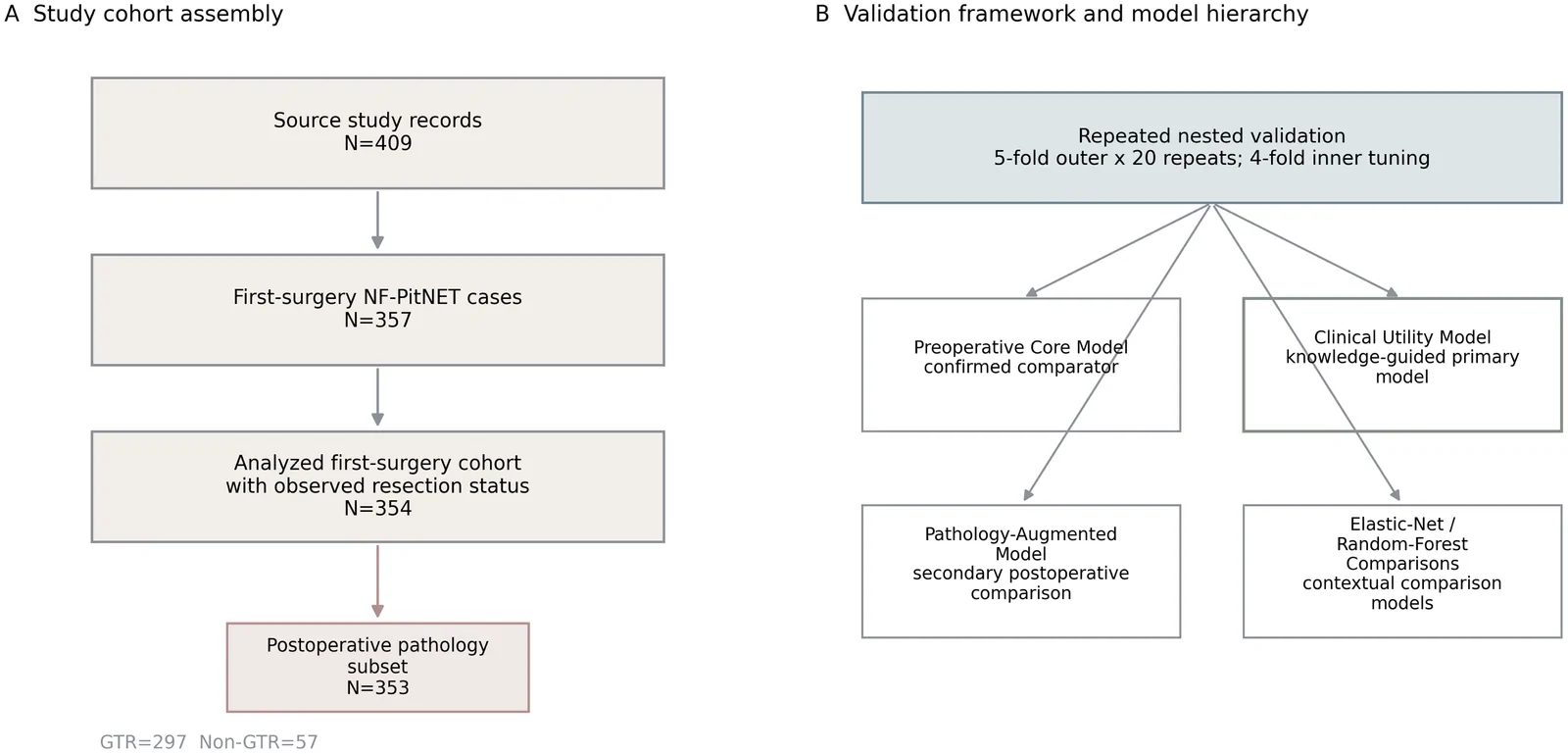
Cohort, knowledge-guided model specification, and internal validation framework for early postoperative residual tumor/non-GTR after first surgery for NF-PitNET. **(A)** shows formation of the frozen 354-patient analysis set and its GTR/non-GTR outcome distribution. Panel **(B)** shows the routine preoperative predictor domains used by the reference strategy, the prespecified transformed terms added by the clinically informed primary strategy, and the distinct secondary roles of postoperative pathology and contextual algorithm benchmarks. In the archived data, invasiveness corresponded to Knosp grades 3-4 and was equivalent to the indicator Knosp >= 3.

This retrospective single-center study analyzed an institutional NF-PitNET cohort derived from patients treated in the Department of Neurosurgery, First Affiliated Hospital of Sun Yat-sen University. The authors’ current affiliation is the Sixth Affiliated Hospital of Sun Yat-sen University. The source cohort comprised 409 eligible NF-PitNET records collected between March 2020 and December 2023 and was previously analyzed for transcription factor-defined clinicopathological subtypes and outcomes (Hu et al., 2026). All 354 patients in the present analysis arose from, and therefore overlapped with, that 409-record source cohort. The present study nevertheless addressed a distinct question by restricting the dataset to first-surgery cases with a documented early postoperative MRI-defined resection status and by developing and internally validating preoperative risk-stratification strategies rather than analyzing pathological subtypes and their outcomes. Of 357 first-surgery patients, 354 were included; three (0.8%) lacked an available resection-status outcome when the analysis dataset was frozen (**Figure 1**). The study was approved by the Clinical Research Ethics Committee of the First Affiliated Hospital of Sun Yat-sen University (approval [2024]576).

The source cohort included clinically nonfunctioning pituitary tumors managed surgically at the First Affiliated Hospital, with perioperative clinical evaluation and postoperative pathological and immunohistochemical assessment sufficient for cohort adjudication. The present analysis used the prespecified first-surgery subset with documented MRI-defined resection status in the frozen analysis dataset. The frozen revision package did not retain the patient-level reasons for the three unavailable outcomes or a complete baseline snapshot for those excluded records; therefore, their characteristics could not be compared reliably with those of the 354 analyzed patients. Endpoint availability was consequently treated as an eligibility requirement and as a potential source of selection bias. All model results, figures, and uncertainty analyses were generated from the internally consistent 354-patient dataset.

The primary endpoint was early postoperative MRI-defined non-GTR, coded dichotomously as GTR versus non-GTR in the archived analytic dataset. Under the source institutional protocol, absence of residual enhancement on postoperative MRI targeted at approximately 3 months was considered GTR, whereas any definite enhancing residual lesion was considered non-GTR. A small minority of records were adjudicated using an earlier postoperative MRI with a definite result that was subsequently supported by follow-up imaging. The frozen endpoint file retained the final binary classification but not exact scan dates, the number examined outside the target window, examination-quality fields, reasons for unavailable outcomes, or a timestamped adjudication trail. A blinded central-read or independent dual-reader log was also not retained. Consequently, neither a timing-distribution summary nor an exclusion sensitivity analysis for earlier or nonstandard imaging could be reconstructed. The endpoint should be interpreted as an institutionally adjudicated early postoperative MRI-based resection-status outcome, not a standardized imaging-core-laboratory endpoint or a long-term residual-growth or recurrence outcome.

All patients underwent preoperative and postoperative 3.0-T MRI. Largest tumor diameter was measured in millimeters on contrast-enhanced T1-weighted MRI across axial, coronal, and sagittal planes and entered as a continuous variable; its transformed term used the natural logarithm of diameter in millimeters. Knosp grade was assessed on contrast-enhanced coronal T1 imaging, recorded on the ordinal 0–4 scale, and entered as the recorded grade. In the archived first-surgery data, the binary invasiveness variable was operationalized as Knosp grades 3–4 and was therefore equivalent to the indicator Knosp ≥3. Age was entered continuously in years. Sex, blurred vision, visual-field defect, and hypopituitarism were entered as binary indicators; the symptom variables were abstracted from the preoperative chart, and hypopituitarism was coded from the institutional endocrine assessment protocol. Macroadenoma, used descriptively rather than as a primary model term, was defined as a largest diameter of at least 10 mm. Predictor definitions, transformations, missing-data handling, and timing of availability are summarized in **Supplementary Table 2**; baseline characteristics are shown in **Table 1**.

**Table 1:** Cohort and preoperative feature summary. Baseline characteristics of the first-surgery NF-PitNET analysis set, stratified descriptively by early postoperative resection outcome.

Characteristic	Overall (N = 354)	Non-GTR (n=57)	GTR (n=297)
Age, years	51 [40-59]	52 [36-59]	51 [40-60]
Largest diameter, mm	25.0 [19.0-30.8]	34.0 [29.0-42.0]	24.0 [19.0-29.0]
Knosp grade	2 [2-3]	3 [2-4]	2 [2-3]
Female sex, n (%)	177 (50.0%)	28 (49.1%)	149 (50.2%)
Blurred vision, n (%)	180 (50.8%)	42 (73.7%)	138 (46.5%)
Visual field defect, n (%)	117 (33.1%)	30 (52.6%)	87 (29.3%)
Hypopituitarism, n (%)	181 (51.1%)	35 (61.4%)	146 (49.2%)
Invasive tumor, n (%)	151 (42.7%)	41 (71.9%)	110 (37.0%)
Macroadenoma, n (%)	351 (99.2%)	57 (100.0%)	294 (99.0%)

Continuous variables are reported as median [IQR] and categorical variables as n (%). The cohort is restricted to first-surgery NF-PitNET cases with observed resection status under the prespecified study definition.

### 2.2 Predictor specification and candidate models

The reference preoperative model was a regularized multivariable logistic regression with eight prespecified input terms available before surgery: age, sex, blurred vision, visual-field defect, hypopituitarism, largest tumor diameter, Knosp grade, and the archived binary invasiveness variable. The primary clinically informed strategy retained those terms and added four transformed terms—natural-log diameter, indicators for Knosp ≥3 and Knosp grade 4, and an invasiveness-by-natural-log-diameter interaction—yielding 12 preoperative input terms before the intercept. Because invasiveness and Knosp ≥3 were equivalent in the archived data, the expanded design intentionally contained overlapping encodings of the same invasion construct. Regularization allowed the model to be fitted, but coefficients of these correlated terms cannot be interpreted as independent effects. Their stability analysis was therefore treated as an exploratory assessment of domain-level structural coherence rather than evidence for individual predictor effects.

The secondary pathology-augmented model added postoperative transcription-factor lineage group and Ki-67 status to the clinically informed feature surface. Pathological classification in the source cohort used hematoxylin-eosin staining, pituitary hormone immunohistochemistry, Ki-67, and the transcription factors PIT1, TPIT, and SF1, categorizing tumors as SF1, TPIT, PIT1, or no distinct/mixed lineage. Ki-67 >= 3% was treated as elevated proliferative activity. Because these variables become available only after surgery, this model was used solely to assess incremental information and was not considered a deployable preoperative model.

All preoperative predictors were complete in the archived 354-patient analysis set. Ki-67 status was missing in one patient (0.3%) in the pathology-augmented layer. Split-wise most-frequent imputation was retained within each training fold for pipelines requiring it, so preprocessing was estimated exclusively from training data and no information crossed into held-out folds. No imputation was required for the primary preoperative comparison itself.

The reference model preserved the previously specified preoperative comparator and its AUROC-based inner-loop selection rule. The clinically informed model was evaluated as a prespecified calibration-oriented extension on the same archived cohort and outer resampling splits. Accordingly, the main comparison is between two modeling strategies that share the same patients and held-out evaluations but differ in both feature representation and inner-loop tuning objective; it should not be interpreted as isolating the causal contribution of the transformed terms alone.

Elastic-net logistic regression and random forest were included as contextual comparison models rather than candidate clinical deployment models. Elastic net tested whether stronger shrinkage and data-driven coefficient selection materially changed performance within a regression framework, whereas random forest provided a nonlinear ensemble benchmark. These models bounded achievable performance on the available evidence surface and did not constitute an exhaustive algorithm search.

### 2.3 Model development, internal validation, and statistical analysis

All candidate models were evaluated under a shared repeated nested cross-validation design with stratified five-fold outer cross-validation repeated 20 times and four-fold inner tuning (**Figure 1**). Within each outer training set, preprocessing and hyperparameter selection were performed using training data only, after which the selected estimator generated probabilities for held-out patients. The reference model tuned the logistic regularization parameter (C ∈ {0.01, 0.1, 1.0, 10.0, 100.0}) against AUROC to preserve its previously specified comparator definition. The clinically informed and pathology-augmented logistic models tuned the same C grid against the negative Brier score to align selection with the prespecified emphasis on probability estimation. Elastic net additionally tuned l1_ratio, and random forest varied tree depth and minimum leaf size within the same nested split hierarchy. The exact candidate values for l1_ratio, tree depth, and minimum leaf size were not retained in the frozen revision package and therefore cannot be reported reliably; this reproducibility limitation is summarized in **Supplementary Table 3**. The different tuning objectives may contribute to the observed calibration difference, and a uniform-tuning sensitivity analysis could not be reconstructed from the archived outputs.

For each patient and model, the 20 held-out probabilities obtained across repeated outer cross-validation were averaged before pooled performance metrics were calculated. Primary measures were AUROC, area under the precision-recall curve, Brier score, calibration intercept, and calibration slope. Calibration intercept and slope were estimated by logistic recalibration of the patient-level averaged out-of-fold probabilities. Additional analyses included calibration plots, decision-curve analysis across thresholds 0.05–0.50, descriptive tertile risk stratification, a threshold-operating grid, risk-group profiles, and bounded subgroup reportability checks. The decision curve did not encode a validated treatment rule. Classification as high risk represented a hypothetical risk-management scenario in which the prediction might prompt more detailed preoperative counseling and/or closer planning of postoperative imaging surveillance. The exploratory 0.05–0.50 range was selected to span low-to-moderate risk tolerances on both sides of the cohort event prevalence (16.1%), rather than from an established clinical protocol. Tertiles were defined from the archived predicted-risk distribution and were not prespecified clinical categories. For the primary comparison, 95% confidence intervals were estimated by stratified patient-level bootstrap of the archived averaged predictions. These intervals condition on the retained prediction set and do not reproduce model refitting or split-to-split variability. For the exploratory stability audit, the clinically informed model was refit across all 100 outer training sets; feature-level coefficients were summarized by their mean and 10th–90th percentiles, and domain-level summaries used mean absolute coefficients and sign consistency. Because related invasion variables overlapped, these summaries were interpreted structurally rather than as independent effects. With 57 events for 12 prespecified input terms, regularization and repeated internal validation reduced but did not eliminate the risk of instability or overfitting. The frozen outputs did not permit a reproducible reduced-model sensitivity analysis using only one invasion representation. Analyses were implemented in Python 3.12.13 using NumPy 2.4.3, pandas 2.3.3, SciPy 1.17.1, and scikit-learn 1.8.0.

## 3 Results

### 3.1 Cohort characteristics and primary model performance

The archived first-surgery analysis set comprised 354 of 357 eligible first-surgery patients; three (0.8%) were excluded because MRI-defined resection status was unavailable in the frozen dataset. The retained case-level information did not support a reliable description of the reasons for unavailability or a baseline comparison with included patients. Among the 354 analyzed patients, 57 (16.1%) had MRI-defined non-GTR and 297 (83.9%) achieved GTR. Cohort assembly is shown in **Figure 1**, and baseline characteristics are summarized in **Table 1**. All performance estimates derive from this same cohort and its outer held-out predictions.

Under the prespecified model-specific tuning strategies, the clinically informed strategy showed only a small change in discrimination: AUROC increased from 0.8022 to 0.8054 and AUPRC from 0.4534 to 0.4603. The corresponding probability estimates had a lower Brier score (0.1100 vs. 0.1480) and calibration intercept and slope closer to their ideal values (0.0256 and 1.0209 vs. -0.3778 and 2.5233; **Table 2**). In the patient-level bootstrap of archived predictions, AUROC was 0.8054 (95% CI, 0.7387-0.8634), Brier score was 0.1100 (0.0970-0.1231), and calibration slope was 1.0209 (0.7510-1.3692) for the clinically informed strategy; corresponding estimates for the reference strategy were 0.8022 (0.7353-0.8604), 0.1480 (0.1399-0.1564), and 2.5233 (1.8471-3.4011). Differences favored the clinically informed strategy for Brier score (-0.0380; 95% CI, -0.0453 to -0.0309) and calibration slope (-1.5024; -2.0531 to -1.0946). These conditional intervals support a difference between the archived prediction sets but do not separate the effects of transformed features from the different inner-loop tuning objectives.

**Table 2:** Comparative performance by analytic role.

Model	AUROC	AUPRC	Brier score	Calibration intercept	Calibration slope
Reference preoperative model (primary comparator)	0.8022	0.4534	0.1480	-0.3778	2.5233
Clinically informed preoperative model (prespecified primary)	0.8054	0.4603	0.1100	0.0256	1.0209
Pathology-augmented model (secondary)	0.8024	0.4614	0.1099	0.0029	1.0044
Elastic-net model (contextual benchmark)	0.8069	0.4851	0.1088	0.1533	1.1110
Random forest model (contextual benchmark)	0.8381	0.5308	0.1004	-0.2059	0.7973

AUROC indicates area under the receiver operating characteristic curve; AUPRC, area under the precision-recall curve. Point estimates are calculated from archived patient-level probabilities averaged across 20 outer repetitions. The reference preoperative model was the primary comparator and was tuned against AUROC. The clinically informed preoperative model was the prespecified primary model, and the pathology-augmented secondary model was tuned against the negative Brier score. Elastic net and random forest were contextual algorithm benchmarks. The table therefore compares complete modeling strategies rather than isolating feature effects. Bootstrap intervals reported in the Results condition on the archived averaged predictions and do not include full model-refitting or split-to-split variability. The random forest achieved the strongest numerical performance but was not the prespecified primary model. The table separates the reference preoperative comparator, the prespecified primary clinically informed model, the secondary pathology-augmented analysis, and contextual algorithm benchmarks under one shared repeated nested internal-validation framework.

### 3.2 Calibration, model-based net benefit, and risk stratification

**Figure 2:**
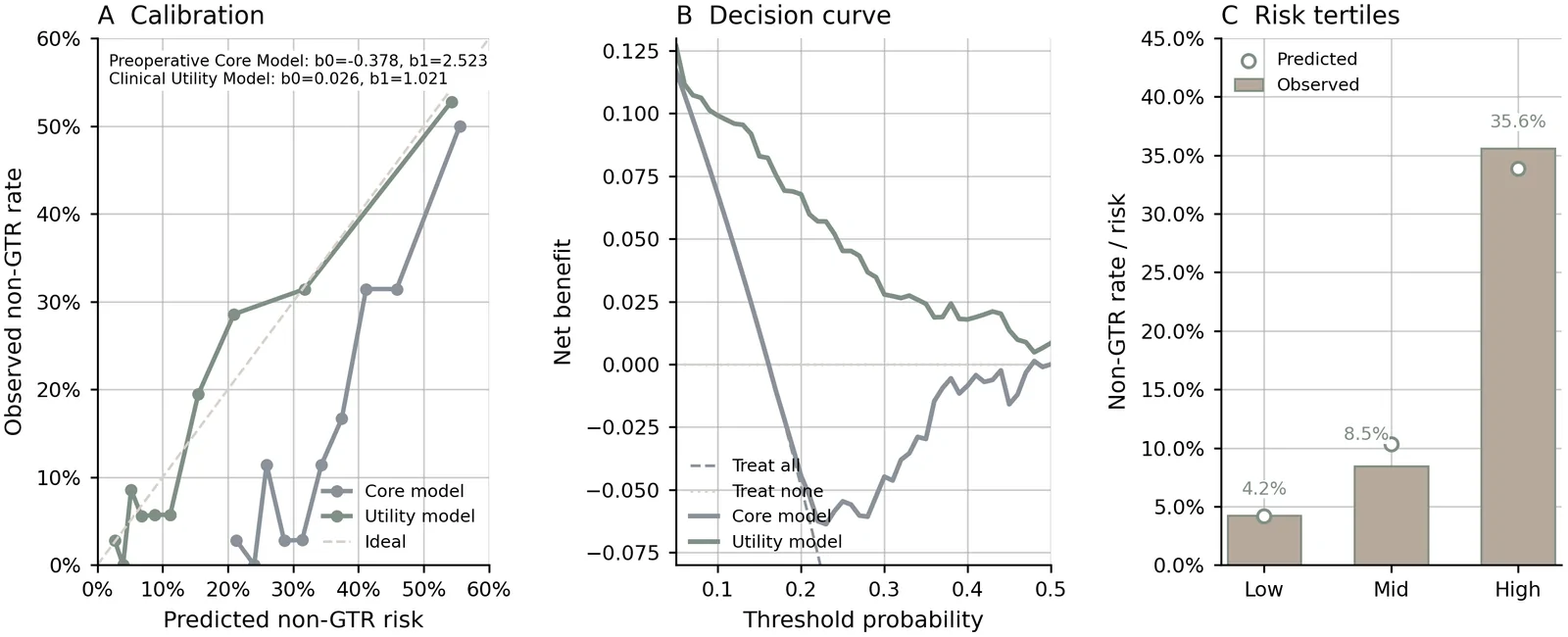
Calibration, decision-curve analysis, and cohort-derived risk tertiles. Panel **(A)** compares agreement between predicted and observed MRI-defined non-GTR risk. Panel **(B)** shows model-based net benefit across an exploratory threshold range of 0.05–0.50; treat all assigns every patient to a hypothetical high-risk management pathway, whereas treat none assigns no patient to that pathway. The analysis was not linked to a validated intervention, and the thresholds are not recommended clinical cutoffs. Panel **(C)** compares mean predicted and observed non-GTR risk across data-defined tertiles, which are descriptive cohort categories rather than externally validated risk groups.

Within the archived cohort, the clinically informed strategy showed closer agreement between predicted and observed risk and greater model-based decision-curve net benefit across the exploratory threshold range of 0.05–0.50 (**Figure 2**). In this analysis, treat all assumes that every patient is assigned to a hypothetical high-risk management pathway and treat none assumes that no patient is assigned to that pathway. The model curve was not linked to a validated intervention; possible future actions could include additional counseling or closer planning of postoperative imaging surveillance. The low-, intermediate-, and high-risk tertiles each contained 118 patients and had observed non-GTR rates of 4.2%, 8.5%, and 35.6%, respectively. These cohort-derived findings describe risk separation and do not establish clinical categories, treatment cutoffs, or clinical effectiveness.

### 3.3 Exploratory coefficient and domain stability

**Figure 3:**
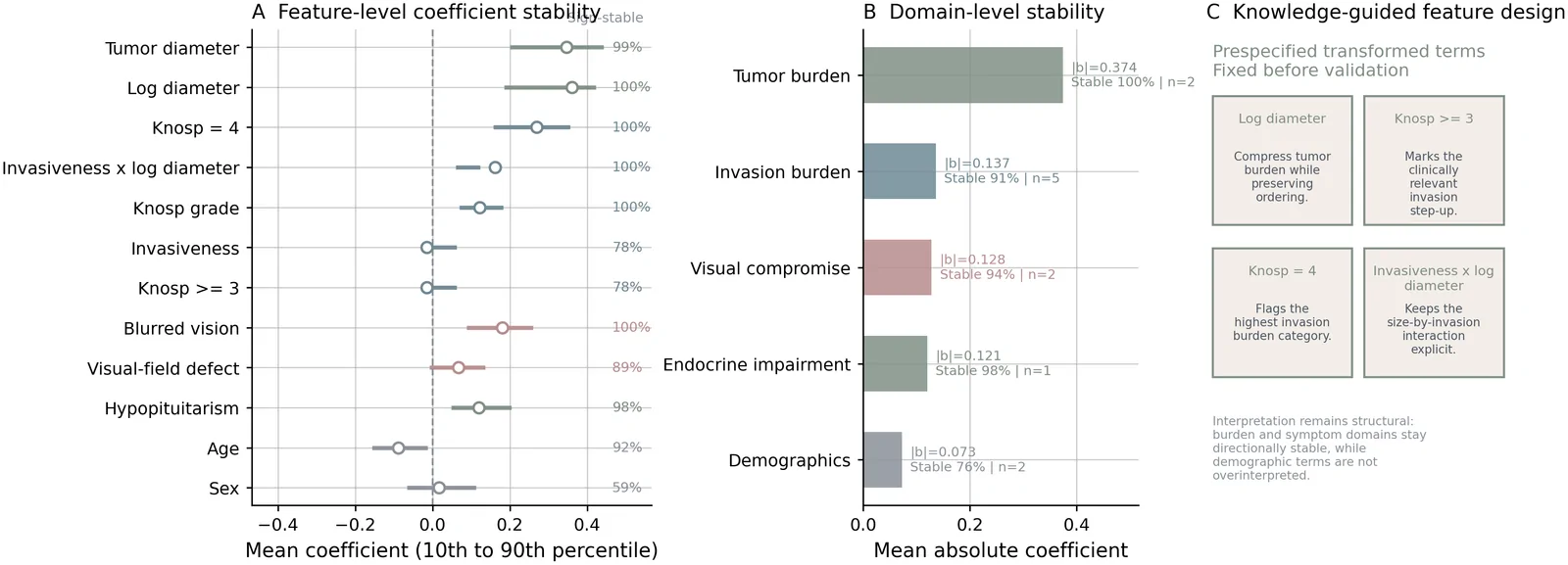
Exploratory coefficient and domain stability of the clinically informed strategy. Panel **(A)** summarizes coefficients across 100 outer-training-set refits using means and 10th-90th percentiles. Panel **(B)** shows domain-level mean absolute coefficients and sign consistency. Panel **(C)** lists the prespecified transformed terms. Because Knosp-related encodings overlap and invasiveness was equivalent to Knosp >= 3 in the archived data, individual correlated coefficients should not be interpreted as independent effects.

Across the 100 outer training sets, tumor-burden terms had the largest domain-level mean absolute coefficient (0.374) and near-complete sign consistency (0.995), while endocrine impairment (0.980), visual compromise (0.945), and invasion-burden features (0.912) were also directionally stable (**Figure 3**). Demographic terms were less stable (0.755). This resampling audit provides some evidence that predictions were repeatedly anchored in the same broad clinical domains. However, the expanded feature set contained 12 input terms for 57 events and included overlapping Knosp-related encodings, with two equivalent binary terms in the archived data. The summaries therefore do not establish stable independent effects for individual correlated coefficients, and a reduced model retaining only one invasion representation could not be reconstructed from the frozen outputs.

### 3.4 Threshold-specific performance and risk-group profiles

**Figure 4:**
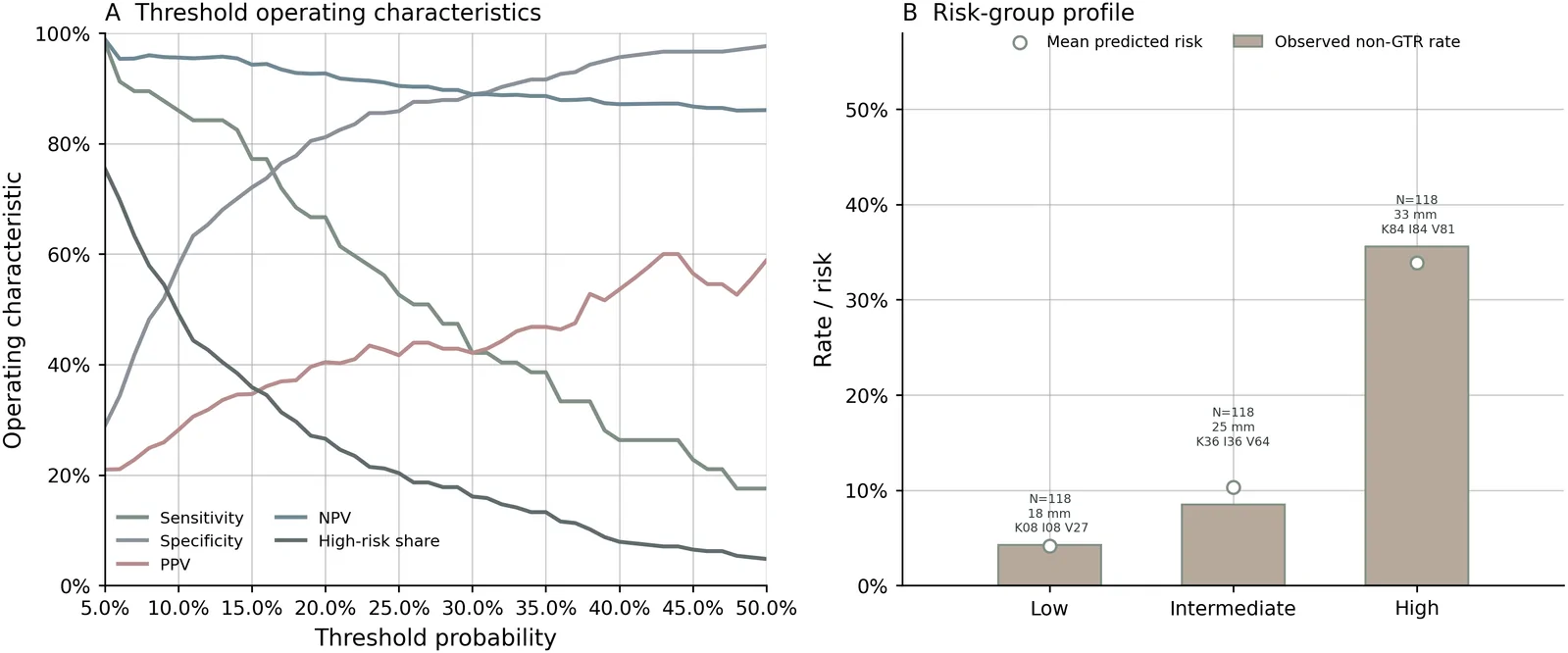
Descriptive threshold operating characteristics and risk-group profiles. Panel **(A)** shows sensitivity, specificity, predictive values, and the proportion classified as high risk across the exploratory threshold range of 0.05–0.50. This range spans thresholds below and above the cohort event prevalence and is not derived from a validated clinical protocol; no threshold is proposed as a treatment or surveillance cutoff. Panel **(B)** describes anatomical and symptomatic profiles across cohort-derived risk tertiles.

The threshold grid provided descriptive operating characteristics across possible risk-management scenarios (**Figure 4**). At a threshold of 0.20, 26.6% of patients would be classified as high risk, with sensitivity 66.7%, specificity 81.1%, positive predictive value 40.4%, and negative predictive value 92.7%. At 0.30, 16.1% would be classified as high risk, with sensitivity 42.1% and specificity 88.9%. These thresholds were selected for illustration rather than optimization and are not recommended treatment cutoffs. Risk-group profiles showed that the high-risk tertile had a median diameter of 33.0 mm, Knosp grade 3-4 in 83.9%, invasiveness in 83.9%, and visual symptoms in 80.5%, compared with 18.0 mm, 7.6%, 7.6%, and 27.1% in the low-risk tertile. The near-universal prevalence of macroadenomas (351/354) limits inference for smaller tumors.

### 3.5 Incremental value of pathology and secondary model comparisons

Adding postoperative pathology variables provided little incremental value relative to the clinically informed preoperative strategy: AUROC changed by -0.0030, AUPRC by +0.0011, and Brier score by -0.0001. Because these variables are unavailable at the preoperative decision point, this result is interpreted as a secondary information-value analysis rather than support for a deployable extension. Pathology and contextual model results are summarized in **Supplementary Figure 2**.

Elastic net produced modest numerical differences, whereas random forest achieved the highest AUROC (0.8381), AUPRC (0.5308), and the lowest Brier score (0.1004), with a calibration slope of 0.7973 (**Table 2**). The clinically informed logistic strategy remained the prespecified primary model because the study question prioritized transparent, knowledge-guided probability estimation at the preoperative decision point. The stronger random-forest results nevertheless indicate that the clinically informed logistic model was not the best-performing algorithm in this cohort and should not be presented as such.

## 4 Discussion

In this internally validated single-center study, the clinically informed strategy was associated primarily with better probability calibration and model-based net benefit rather than materially higher discrimination. The lower Brier score, calibration slope nearer 1.0, decision-curve pattern, and clearer tertile separation support a calibration-centered interpretation within the study cohort. However, the reference and clinically informed models used different inner-loop tuning objectives. The results therefore compare complete prespecified modeling strategies and do not prove that the knowledge-guided transformations alone caused the calibration improvement.

The intended future use is preoperative discussion before first surgery, not autonomous decision-making. After independent validation, an estimated probability might help explain the likelihood of MRI-defined non-GTR, align expectations about achievable resection, and support planning of postoperative MRI surveillance when anatomy is unfavorable. In the present study, however, the decision curve represents only model-based net benefit under hypothetical risk-management scenarios. Neither the illustrative threshold grid nor the cohort-derived tertiles should determine surgery, adjuvant therapy, counseling content, or surveillance intensity without external validation and prospective clinical-impact evaluation.

This framing also locates the paper relative to the structural resectability literature. Existing surgical series and grading systems consistently center anatomy-driven complexity, especially tumor burden, Knosp grade, and invasion burden, whether in single-center reports or simplified tools such as Zurich Pituitary Score, HACKD, and the original-to-modified TRANSSPHER grading line (Juraschka et al., 2014; Mooney et al., 2019; Staartjes et al., 2020; Perez-Lopez et al., 2021; Castle-Kirszbaum et al., 2022; Fiore et al., 2025). More recent anatomy-limit and landmark-based nomograms extend that structural route and can report strong internal discrimination, calibration, and DCA, while NFPA-specific radiological scores, disease-matched giant nonfunctioning PitNET predictive modeling from structured multi-planar variables, automated Zurich-score grading, and newer giant-adenoma multivariate morphologic models show that the same line is still being refined and operationalized inside disease-matched structural frames (Martorell-Llobregat et al., 2023; Shen et al., 2024; Da Mutten et al., 2025; Giardini et al., 2025; Patel et al., 2025a; Ozaydin et al., 2026). Yet those studies still remain closer to resectability prediction or score execution than to calibrated residual-risk estimation within a prespecified analytic design. Against this background, our study kept the preoperative feature set and validation design fixed and asked whether calibrated residual-risk probabilities and threshold-specific net benefit could be improved within an interpretable logistic framework. Using NF-PitNET terminology while bridging to older pituitary adenoma language also keeps the manuscript aligned with current WHO nomenclature and with the older surgical comparator literature (Asa et al., 2022).

This manuscript should also be interpreted alongside higher-capacity pituitary prediction studies. Machine-learning studies have already explored early outcomes or gross-total-resection classification after pituitary surgery, and one later study externally validated preoperative pituitary-surgery prediction models across multiple postoperative endpoints (Hollon et al., 2018; Staartjes et al., 2018; Zanier et al., 2023). The comparator neighborhood now also includes radiomic GTR/consistency prediction and CNN-based EOR regression from preoperative MRI, and a 2025 systematic review/meta-analysis suggests that ML-based remission prediction after pituitary surgery is also expanding in the literature, but on endpoint-mixed postoperative cohorts rather than prespecified preoperative residual-risk populations (Cerny et al., 2025; Mohammadzadeh et al., 2025; Patel et al., 2025b). A separate 2026 meta-analysis reported strong pooled accuracy for ML-based preoperative consistency prediction but again highlighted the need for standardized imaging and external validation before clinical translation (Hajikarimloo et al., 2026). A pituitary-surgery ML systematic review and a more recent radiomics review both suggest that the field remains methodologically heterogeneous, incompletely calibrated, and sparsely externally validated (Rech et al., 2023; Agosti et al., 2025). Read against newer reporting and bias-appraisal frameworks such as TRIPOD+AI and PROBAST+AI, that combination is better treated as translational caution than as immediate clinical readiness (Collins et al., 2024; Moons et al., 2025). Although the random forest comparison achieved the highest numerical discrimination in this cohort, its lower interpretability and less favorable calibration slope make it better suited as a contextual performance bound than as the primary clinical model. The pathology comparison is likewise contextual because those markers sit outside the preoperative decision point targeted by this manuscript.

Several limitations are important. First, this retrospective single-center study included only 57 non-GTR events for a clinically informed surface of 12 input terms, including correlated invasion encodings. Regularization, repeated nested cross-validation, and the 100-refit coefficient audit reduce but do not eliminate model instability or overfitting, and a reduced-model sensitivity analysis retaining only one invasion representation could not be reconstructed. Second, 351 of 354 patients had macroadenomas, leaving essentially no support for smaller tumors. Third, the institutionally adjudicated MRI endpoint lacked a retained timestamped adjudication dataset, examination-quality fields, and blinded central or dual-reader review. The exact scan-timing distribution and the number assessed outside the target window could not be reconstructed, precluding a timing-window sensitivity analysis. Fourth, three first-surgery cases lacked a frozen resection-status outcome; their reasons for missingness and baseline characteristics were not retained sufficiently for comparison, so selection bias cannot be excluded. Fifth, the reference and clinically informed strategies used different tuning metrics, and a uniform-metric sensitivity analysis was unavailable. The exact elastic-net and random-forest candidate grids were also not retained. Bootstrap intervals were calculated from archived patient-level averaged predictions and did not include complete model-refitting or split-to-split variability; repeat-level performance traces were unavailable. Finally, decision curves, thresholds, tertiles, subgroup summaries, and the random-forest benchmark are exploratory, cohort-derived evaluations. They do not establish transportability, deployment readiness, a clinical cutoff, or improved patient outcomes.

In conclusion, this single-center internal-validation study found that a clinically informed preoperative logistic strategy was associated with better calibration and model-based net benefit than the preserved reference strategy, with little change in discrimination and little incremental information from postoperative pathology. These results apply to the complete modeling strategies evaluated in this cohort; they do not isolate the effect of transformed predictors or establish a deployable clinical tool. Independent validation using standardized postoperative MRI, uniform-tuning and reduced-model sensitivity analyses, and prospective evaluation of any counseling or surveillance pathway are required before clinical use.

## Data Availability

The raw data supporting the conclusions of this article will be made available by the authors, without undue reservation.
