# Supplementary material for: Clinically informed preoperative risk stratification for MRI-defined non gross-total resection in nonfunctioning pituitary neuroendocrine tumors: a single-center internal validation study
Source: Front Physiol. 2026 Sep 7;17:1948507. doi: 10.3389/fphys.2026.1948507 (PMC13590360; doi:10.3389/fphys.2026.1948507)
Supplement: Supplementary file 1 [file SupplementaryFile1.docx]

**Supplementary Material**

This file contains Supplementary Tables S1–S3 and Supplementary Figures S1–S2.

**Supplementary Table S1. Bounded subgroup support for the clinically informed preoperative model.** The non-large-tumor slice is included only to make the sparse-data boundary explicit; all other rows are contextual stability checks within the study cohort.

| **Domain** | **Slice** | **Cases** | **Events** | **AUROC** | **Brier** | **Cal. slope** | **Note** |
| --- | --- | --- | --- | --- | --- | --- | --- |
| Tumor size | Non-large | 3 | 0 | NA | NA | NA | Insufficient support: n=3, 0 events. |
| Tumor size | Large | 351 | 57 | 0.8035 | 0.1110 | 1.0181 | Reportable; calibration near ideal. |
| Knosp | 0-2 | 203 | 16 | 0.7433 | 0.0686 | 1.0894 | Reportable; low event burden. |
| Knosp | 3-4 | 151 | 41 | 0.7608 | 0.1658 | 0.9778 | Reportable; calibration near ideal. |
| Invasiveness | Non-invasive | 203 | 16 | 0.7433 | 0.0686 | 1.0894 | Reportable; lower-risk slice. |
| Invasiveness | Invasive | 151 | 41 | 0.7608 | 0.1658 | 0.9778 | Reportable; higher-risk slice. |
| Visual symptoms | No symptoms | 152 | 14 | 0.8659 | 0.0663 | 1.8000 | Caution: calibration slope unstable. |
| Visual symptoms | Symptoms | 202 | 43 | 0.7503 | 0.1429 | 0.8484 | Reportable; less stable than full cohort. |

**Note:** This supplementary table is descriptive and boundary-setting rather than confirmatory. NA indicates that the estimate was not reportable because no events occurred in the subgroup.

**Supplementary Table S2. Predictor definitions, coding, missing-data handling, and timing of availability.**

| **Predictor / term** | **Model role** | **Coding, unit, or transformation** | **Missing-data handling** | **Availability** |
| --- | --- | --- | --- | --- |
| Age | Reference and clinically informed | Continuous; years | Complete in primary cohort | Preoperative |
| Sex | Reference and clinically informed | Binary indicator; female vs. male | Complete in primary cohort | Preoperative |
| Blurred vision | Reference and clinically informed | Binary; present vs. absent from chart | Complete in primary cohort | Preoperative |
| Visual-field defect | Reference and clinically informed | Binary; present vs. absent from chart | Complete in primary cohort | Preoperative |
| Hypopituitarism | Reference and clinically informed | Binary; present vs. absent under institutional endocrine assessment | Complete in primary cohort | Preoperative |
| Largest tumor diameter | Reference and clinically informed | Continuous; millimeters on contrast-enhanced T1 MRI | Complete in primary cohort | Preoperative |
| Knosp grade | Reference and clinically informed | Ordinal 0–4; entered as recorded grade | Complete in primary cohort | Preoperative |
| Invasiveness | Reference and clinically informed | Binary; Knosp grades 3–4 vs. 0–2; equivalent to Knosp ≥3 in archived data | Complete in primary cohort | Preoperative |
| ln(diameter) | Clinically informed transformed term | Natural logarithm of largest diameter in millimeters | Derived after training-fold preprocessing | Preoperative |
| Knosp ≥3 | Clinically informed transformed term | Binary indicator; equivalent to invasiveness in archived data | Derived from complete Knosp grade | Preoperative |
| Knosp grade 4 | Clinically informed transformed term | Binary indicator; grade 4 vs. grades 0–3 | Derived from complete Knosp grade | Preoperative |
| Invasiveness × ln(diameter) | Clinically informed transformed term | Product of binary invasiveness and natural-log diameter | Derived from complete inputs | Preoperative |
| Transcription-factor lineage | Pathology-augmented secondary model | Categorical: SF1, TPIT, PIT1, or no distinct/mixed lineage | Training-fold preprocessing | Postoperative |
| Ki-67 ≥3% | Pathology-augmented secondary model | Binary indicator of elevated proliferative activity | One missing value; most-frequent imputation within each training fold | Postoperative |

**Note:** The primary preoperative comparison required no imputation. Invasiveness and Knosp ≥3 are explicitly reported as equivalent archived encodings and should not be interpreted as independent anatomical constructs.

**Supplementary Table S3. Model roles and tuning information retained in the frozen revision package.**

| **Model** | **Analytic role** | **Retained candidate hyperparameters** | **Inner-loop objective** | **Revision-package limitation** |
| --- | --- | --- | --- | --- |
| Reference logistic | Primary comparator | C ∈ {0.01, 0.1, 1.0, 10.0, 100.0} | AUROC | Complete retained grid |
| Clinically informed logistic | Prespecified primary | C ∈ {0.01, 0.1, 1.0, 10.0, 100.0} | Negative Brier score | Complete retained grid |
| Pathology-augmented logistic | Secondary information-value analysis | C ∈ {0.01, 0.1, 1.0, 10.0, 100.0} | Negative Brier score | Complete retained grid |
| Elastic net | Contextual benchmark | C plus l1_ratio | Archived benchmark selection | Exact l1_ratio candidate values were not retained |
| Random forest | Contextual benchmark | Tree depth and minimum leaf size | Archived benchmark selection | Exact candidate values were not retained |

**Note:** The unavailable elastic-net and random-forest candidate values are reported as a reproducibility limitation rather than reconstructed from assumptions. These algorithms remain contextual benchmarks and do not determine the primary study conclusion.

# Supplementary Figures

**Supplementary Figure S1. Subgroup reportability boundaries for the clinically informed preoperative model.** Subgroup reporting is bounded rather than universal: the non-large-tumor subgroup is explicitly marked as insufficient support, and the remaining slices are presented as contextual stability checks within the study cohort.

**Supplementary Figure S2. Pathology added-value and contextual model comparisons around the clinically informed preoperative model.** Pathology added little incremental value, and comparison-model performance ceilings did not change the primary claim centered on the clinically informed preoperative model.
